# Efficient hash tables for network applications

**DOI:** 10.1186/s40064-015-0958-y

**Published:** 2015-05-15

**Authors:** Thomas Zink, Marcel Waldvogel

**Affiliations:** Distributed Systems Laboratory, University of Konstanz, Konstanz, Germany

## Abstract

Hashing has yet to be widely accepted as a component of hard real-time systems and hardware implementations, due to still existing prejudices concerning the unpredictability of space and time requirements resulting from collisions. While in theory perfect hashing can provide optimal mapping, in practice, finding a perfect hash function is too expensive, especially in the context of high-speed applications.

The introduction of hashing with multiple choices, *d-left hashing* and probabilistic table summaries, has caused a shift towards deterministic DRAM access. However, high amounts of rare and expensive high-speed SRAM need to be traded off for predictability, which is infeasible for many applications.

In this paper we show that previous suggestions suffer from the false precondition of full generality. Our approach exploits four individual degrees of freedom available in many practical applications, especially hardware and high-speed lookups. This reduces the requirement of on-chip memory up to an order of magnitude and guarantees constant lookup and update time at the cost of only minute amounts of additional hardware. Our design makes efficient hash table implementations cheaper, more predictable, and more practical.

## Introduction

Efficient hashing in network applications is still a challenging task, because tremendously increasing line speed, demand for low power consumption and the need for performance predictability pose high constraints on data structures and algorithms. At the same time, memory access speed has almost stayed constant, especially because of the latency and waiting time between sequential repeated accesses. Hashing has yet to be widely accepted as an ingredient in hard real-time systems and hardware implementations, as prejudices concerning the unpredictability of size and time requirements due to collisions still persist.

Modern approaches make use of multiple choices in hashing (Broder and Mitzenmacher 2001; Vöcking 2003) to improve load and the number of memory accesses. Unfortunately, *d-ary* hashing requires *d* independent parallel lookups. To mitigate the need for high parallelism, table summaries (Kirsch and Mitzenmacher 2008; Song et al. 2005), based on (counting) Bloom filters (Bloom 1970; Fan et al. 1998) and derivates, further reduce the number of table accesses to one with high probability (w.h.p.) at the cost of fast but expensive on-chip memory (SRAM). The summaries allow set membership queries with a low false positive rate and some approaches also reveal the correct location of an item if present.

Although these improvements address space and time requirements, they come at a high price. SRAM is extremely expensive and, while external DRAM can be shared, it must be replicated for every network processor. In addition, numerous networking applications compete for their slice of this precious memory. For many - like socket lookups, Layer-2 switching, packet classification and packet forwarding - tables and their summaries tend to grow extremely large, up to the point where providing enough SRAM is not applicable. Perfect hashing, on the other hand, can lead to a near perfect match (Hagerup and Tholey 2001) but only works on static sets, does not allow updates and requires complex computations.

The options for a network application designer are grim. With millions of lookups per second, even the most improbable worst-case is likely to happen, slowing down the entire application and leading to packet loss and network congestion. Naive hash tables are too unpredictable and yield too many collisions. *d*−*ary* hashing requires high parallelism to minimize sequential lookups. Expensive SRAM-based table summaries optimize the average case performance but still require multiple lookups in the worst case. Perfect hashing can potentially guarantee a perfect match and a constant lookup performance but requires a static set. To be fully accepted in practical network applications hashing needs to guarantee constant lookup performance, require minimal on-chip memory, and allow regular updates.

We propose mechanisms to construct an improved data structure which we name *Efficient Hash Table (EHT)*, where efficient relates to both on-chip memory (SRAM) usage and lookup performance. The design aggressively reduces the amount of bits per item needed for the on-chip summary, guarantees a constant lookup time and still delivers adequate update performance for most applications, except those that require real-time updates. To the best of our knowledge, the EHT is the only data structure offering these characteristics.

Previous approaches suffer from the need for full generality. Careful observation of network applications reveals certain degrees of freedom which can be exploited to achieve significant improvements. These observations lead to the following four key ideas:

- The update and lookup engines can be separated. The on-chip summary need not to be exact.
- The summary’s false positive rate can be ignored, it is irrelevant in respect to lookup performance.
- The summary can be de/compressed in real time.
- The load of a bucket can potentially be larger than one without increasing memory accesses.

In concert, these concepts reduce SRAM memory size up to an order of magnitude, but they can also be applied and configured individually depending on the target application.

The rest of this paper is organized as follows. Section 2 discusses related work with Section 2.1 reviewing hash table summaries is greater detail. Section 3 introduces the *Efficient Hash Table* and presents an overview. Section 4 shows how to separate the update and lookup engines. Section 5 discusses the effect of the false positive rate on the EHT. Section 6 presents multiple compression schemes to improve SRAM memory footprint. Section 7 shows how to optimize bucket loads. The results are evaluated and discussed in Section 8. Finally, the paper concludes in Section 9.

## Related work

A hash function *h* maps items of a set *S* to an array of buckets *B*. Their natural applications are hash tables, or dictionaries, that map keys to values. In theory, a *perfect hash function* that is injective on *S* (Hagerup and Tholey 2001), could map *n* items to *n* buckets. While perfect hashing for static sets is relatively easy (Fredman et al. 1984), finding a suitable hash function that requires constant space and time to perform the mapping of a dynamic set is infeasible in practice. As a result, hashing has to deal with collisions, where multiple items are hashed into the same bucket. Naive solutions anchor a linked list or an array of items to the overflown bucket or probe multiple buckets according to a predefined scheme. The need for collision resolution led to the persisting myth that hashing has unpredictable space/time requirements.

Dietzfelbinger et al. 1994 extended the scheme of Fredman et al. 1984 to store dynamic sets. Their dynamic perfect hashing resolves collisions by random selection of universal hash functions (Carter and Wegman 1977) for a second-level hash table.

Azar et al. 1994 observed, that by allowing more possible destinations for items and choosing that destination with lowest load, both, the average as well as the upper bound load, can be reduced exponentially. This effect became popular as the “power of two choices”, a term coined by Mitzenmacher in (Mitzenmacher 1996). Vöcking 2003 achieved further improvements by introducing the “always-go-left” algorithm, where the items are distributed asymmetrically among the buckets. Broder and Mitzenmacher 2001 suggest using multiple hash functions to improve the performance of hash tables. The *n* buckets of the table are split into *d* equal parts imagined to run from left to right. An item is hashed *d* times to find the *d* possible locations. It is then placed in the least loaded bucket. Ties are broken by going left (*d-left hashing*). A lookup requires examining the *d* locations. Since the *d* choices are independent, lookups can be performed in parallel or pipelined. A survey of multiple-choice hashing schemes and their applications can be found in (Mitzenmacher 2001a).

*Bloom Filters* (Bloom 1970) represent set memberships of a set *S* from a universe *U*. They allow false positives, that is, they can falsely report the membership of an item not in the set, but never return false negatives. Basically, a Bloom filter is a bit array of arbitrary length *m* where each bit is initially cleared. For each item *x* inserted into the set, *k* hash values {*h*_0_,…,*h*_*k*−1_} are produced while $\forall {h} \in \mathbb {N} : 0 \leq h < m$. The bits at the *k* corresponding positions are then set. A query for an item *y* just checks the *k* bits corresponding to *y*. If all of them are set, *y* is reported to be a member of *S*. A false positive occurs, if all bits corresponding to an item not in the set are 1. The probability that this happens depends on the number of items *n* inserted, the array length *m*, and the number of hash functions *k* as shown in Eq. 1.

(1) $$ \epsilon = \left(1-\left(1-\frac{1}{m}\right)^{kn}\right)^{k}.  $$

The major drawback of Bloom filters is that they do not allow deletions. Fan et al. 1998 addressed this issue by introducing a *counting Bloom filter (CBF)*. Instead of a bit array, CBF maintains an array of counters *C*={*ς*_0_,…,*ς*_*m*−1_} to represent the number of items that are hashed to its cells. Insertions and deletions can now be handled easily by incrementing and decrementing the corresponding counters. Later, Bonomi et al. presented an improved version of CBF based on *d-left hashing* (Bonomi et al. 2006).

In (Mitzenmacher 2001b) Mitzenmacher proposes arithmetic coding for Bloom filters used for exchanging messages (web cache information) in distributed systems. Recently, Ficara et al. 2008 introduced a compression scheme for counting Bloom filters based on Huffman coding named *MultiLayer Compressed Counting Bloom Filter (ML-CCBF)*. The compressed counters are stored in multiple layers of bitmaps. Indexing requires perfect hash functions since collisions must be avoided. The structure provides near optimal encoding of the counters but retrieval is extremely expensive. The authors propose splitting the bitmaps into equal sized blocks and using an index structure to lower the cost of a counter lookup.

Bloom filters have since gained a lot of attention especially in network applications (Broder and Mitzenmacher 2002). Today, Bloom filters can be used as histograms (Cohen and Matias 2003) and represent arbitrary functions (Chazelle et al. 2004). In 2005 Song et al. 2005 suggested using Bloom filters as a hash table summary. This idea was later refined in (Kirsch and Mitzenmacher 2005). Bloom filter-based summaries are also used for minimal perfect hashing (Lu et al. 2006).

### 2.1 Review of hash table summaries

Our work is based on the schemes presented by Song et al. 2005 and Kirsch and Mitzenmacher 2005, which we will now review for completeness.

Song et al. 2005 presented a new hash table design, named *Fast Hash Table*, based on hashing with choices and counting Bloom filter summaries that targets hardware implementations and provides fast lookups by utilizing on-chip memory to optimize performance. Their scheme eliminates the need for parallel lookups usually required by multiple-choice hashing. Each *b*-bit counter (*b*=3) in CBF summary corresponds to a bucket in the hash table and represents the number of items hashed into it. Note, that if *b* is small, the probability of counter overflows can’t be neglected. Song et al. proposed using a small CAM for overflown counters. There are a total of *m* counters (and buckets) where *m* is calculated using Eq. 2.

(2) $$ m_{\textrm{FHT}} = 2^{\lceil \log{c\ n}\rceil}  $$

The constant *c* needs to be sufficiently large to provide low false positive and collision probabilities. It is set to 12.8 which is considered optimal. *k* independent hash functions, where *k* is derived by Eq. 3, are used to index both CBF and the hash table.

(3) $$ k = \left\lceil \frac{m}{n} \ln{2} \right\rceil  $$

**Figure 1:**
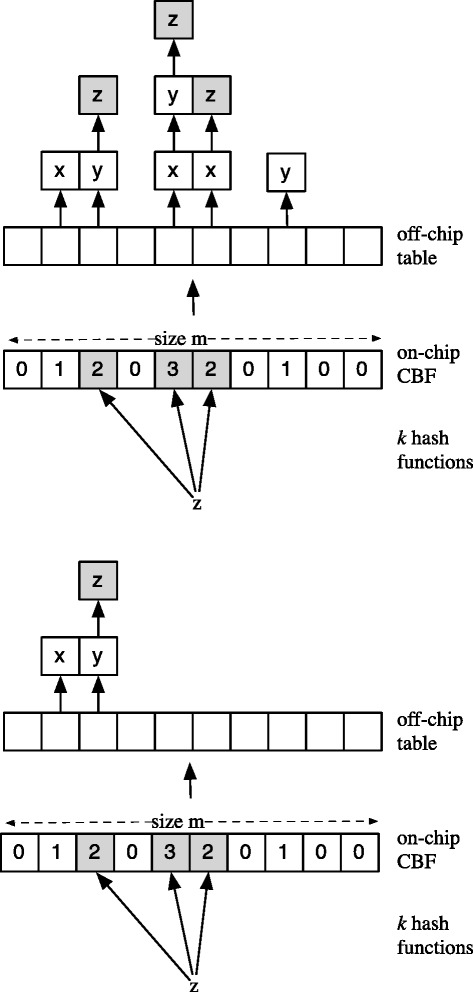
The two fast hash tables. The Basic FHT (top) replicates every item. The Pruned FHT (bottom) only keeps the ‘left’most (‘Left’ refers to the table entry with the least index).

The *Basic Fast Hash Table (BFHT)* simply replicates all inserted items to all *k* locations in the table and increments the counters. As an improvement the table can be *pruned* leading to a *Pruned Fast Hash Table (PFHT)*. All replicas are removed except for the leftmost with the lowest counter value (Figure 1). A lookup only requires examining the least loaded bucket, i.e., the one with the lowest counter value. While pruning improves lookup time by reducing bucket loads, updates require an additional offline BFHT since items need to be relocated when their associated counters change.

Following Eq. 2 the total amount of bits *β* needed for the on-chip summary is dependent on the number of items and defined as

(4) $$ \beta_{\textrm{FHT}} = 2^{\lceil \log{c\ n}\rceil} \cdot b  $$

**Figure 2:**
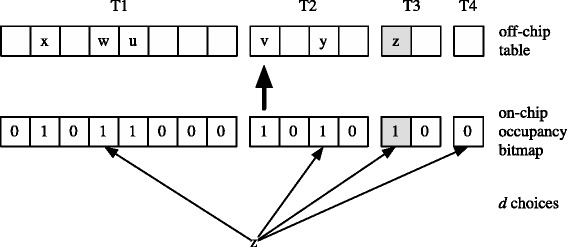
Multilevel hash table with on-chip occupancy bitmap.

The rather high requirement of SRAM has later been addressed by Kirsch and Mitzenmacher 2005. Their key idea is to separate the hash table from its summary to allow individual optimizations. They propose using a *Multilevel Hash Table (MHT)* (Broder and Karlin 1990) consisting of *d*= log log*n*+1 individual tables geometrically decreasing in size. An occupancy bitmap is kept in on-chip memory that allows efficient queries for empty buckets (Figure 2).

The bitmap requires a number of bits equal to the number of buckets *m* which is defined as

(5) $$ \beta_{\textrm{MHT}} = m_{\textrm{MHT}} = \sum_{i=1}^{d} \left(c_{1} \cdot c_{2}^{i-1} \cdot n\right)  $$

with the constants *c*_1_, *c*_2_ chosen such that *c*_1_>1, *c*_2_<1, *c*_1_*c*_2_>1. Considering only the number of buckets per item the equation boils down to

(6) $$ \beta_{\textrm{MHT'}} = m_{\textrm{MHT'}} = c \cdot n  $$

The authors argue that *c*=6 buckets per item suffice. Later in (Kirsch and Mitzenmacher 2010) the authors refine the MHT by limiting the amount that items are allowed to be moved during insertions. In the most aggressive optimization schemes this can reduce the number of buckets per item to *c*<2 for *n*=10^4^ at the cost of additional complexity. Note, that this does not affect the on-chip requirements of the MHT summaries, since they are deliberately separated from the actual hash table and their size only depends on the number of items. It has, however, an impact on the size of the occupancy (and deletion) bitmap.

**Figure 3:**
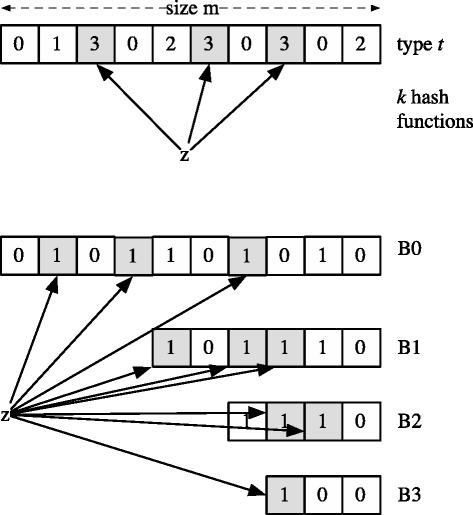
Single filter (SF) and multiple Bloom filter (MBF) summaries. The SF is a single Bloomier filter representing the type of an item. The MBF is an array of Bloom filters decreasing in size.

Following Song et al. to eliminate parallel lookup overhead, Kirsch and Mitzenmacher present three summary structures, the *interpolation search (IS)*, *single filter (SF)* and *multiple Bloom filter (MBF)* summaries. Since IS is not applicable in our targeted environment we will cover only the latter two summaries which are based on Bloom filters. They are depicted in Figure 3.

The SF summary is a single Bloomier filter (Chazelle et al. 2004) representing the type *t* of an item where *t* corresponds to the sub-table of the MHT where the item is located. In addition to false positives, it can also return type failure. To keep the probability small the filter must be sufficiently large. The number of cells *m* is defined as

(7) $$ m = n\ \log n  $$

With log log log*n* bits per cell the number of bits needed is

(8) $$ \beta_{\text{SF}} = n\ \log n\ (\log \log \log n)  $$

The MBF summary is constructed of an array of Bloom filters *B*={*B*_0_,...,*B*_*t*−1_}. Each filter *B*_*i*_ represents the set of items with type of at least *i*+1. Thus, a false positive on *B*_*i*_ is equal to a type *i* failure. This leads to the need of extremely small false positive probabilities to guarantee successful lookup. For a well designed MHT the number of bits the MBF requires is

(9) $$ \beta_{\textrm{MBF}} = n\ \log n  $$

Both, the SF and MBF summaries, support only inserts. To allow deletions, considerably more effort is required. Kirsch and Mitzenmacher suggest two approaches. For *lazy deletions* a deletion bitmap is kept alongside the occupancy bitmap in on-chip memory with one bit for every bucket in the MHT. On deletion, the corresponding bit is set to 1. During lookup, items in buckets that have a set deletion bit are ignored. The *counter based deletions* add counters to the SF and MBF summaries to keep track of the actual number of items. The authors do not suggest specific values for the counter width nor provide evaluation. They state however, that a counting MBF requires about 3.3 times more space than a simple MBF, that is

(10) $$ \beta_{\textrm{MCBF}} = 3.3 \cdot n \log n  $$

With *d* choices and *υ* wide counters the modified SF requires

(11) $$ \beta_{\textrm{SFc}} = \upsilon \cdot d \cdot n\ \log n  $$

bits.

A predecessor to the MHT is the *Segmented Hash Table* (Kumar and Crowley 2005) that also divides the hash table into multiple segments. Unlike the MHT, however, segments are equal sized. Each segment uses a Bloom filter to support membership queries for an item. The false positive probability needs to be extremely low to prevent sequential or parallel probing of multiple segments. A novel *selective filter insertion* algorithm minimizes the number of non-zero counters by selecting that segment for insertion that leads the most empty counters. Thus false positive probability can be reduced. The authors argue that 16 bits per item of on-chip memory and 16 or more segments suffice to provide good performance. To also support deletions, an additional counting Bloom filter must be kept offline.

The authors later refine segmented hashing in (Kumar et al. 2008) which they name *peacock hash*. As with the MHT the idea is to have multiple segments that geometrically decrease in size according to a so called *scaling factor*. Each table, except the biggest main table, has an on-chip Bloom filter for membership queries. When an item is searched the filters of the subtables are queried. If lookup is unsuccessful, the main table is probed. Again, the false positive probability needs to be extremely low to prevent multiple table accesses. With a scaling factor of 10 (each successive table has a size of 10*%* of the former) and following the observations in (Kumar and Crowley 2005), about 2 bits per item are needed for the on-chip Bloom filters.

The problem of non-deterministic lookup performance is addressed in (Ficara et al. 2009). Here each item is associated with a *fingerprint* that is cut into chucks and stored in a small *discriminator* table. This table is used to index the main table and is stored on-chip. Fingerprints must be unique to prevent collisions. A genetic algorithm is suggested to find the perfect mapping. The authors show that a discriminator table with 4 bits per item can be found in a reasonable amount of time. While it is possible to “build a perfect match […] with fewer [2] bits per item […] the effort […] greatly exceeds the advantages.” ((Ficara et al. 2009), p.141.) Also, being a perfect hashing scheme, it works only on static sets and the discriminator table can only be built if the set of items is known a priori.

Recently, the construction of collision-free hash tables has been discussed in (Li and Chen 2013). The authors proposed the addition of an on-chip *summary vector* between the Bloom filter summary and the hash table. This summary vector allows deterministic lookup at the cost of additional on-chip memory.

## Efficient hash tables

We improve upon previously suggested solutions and design an *Efficient Hash Table (EHT)*. The EHT reduces on-chip memory requirements, provides a constant lookup performance and thus predictability, and, unlike comparable perfect hashing schemes, it is still updatable and works with dynamic sets.

This is achieved by exploiting degrees of freedom present in many lookup intensive applications. Previous work has shown that flexibility must be bought with on-chip memory. By completely separating updates from lookups, the lookup engine can be optimized independently and precious on-chip memory saved. The offline update engine precomputes all changes on the online structures and only writes necessary changes (Section 4). Further, we observe that the summary’s false positive rate is irrelevant in respect to lookup performance. By ignoring the false positive rate, the length of the on-chip summary can be aggressively reduced (Section 5). However, this leads to an increased rate of collisions and multiple items compete for the same bucket. In order to prevent multiple lookups, clever fingerprinting and verification can reduce the sizes of items and allow multiple entries per bucket (Section 7). To further reduce the on-chip summarie’s memory cost, we suggest a Huffman compression scheme suitable for real-time (de)compression (Section 6).

The following sections explain the different components in great detail. We start by separating the update and lookup engines in Section 4. Next, we explore the effect of the false positive rate on expected counter values and number of collisions - bucket load - in Section 5. Then we show how to further reduce on-chip memory cost by using Huffman compressed Bloom filter summaries (Section 6). Finally, Section 7 shows how to achieve a guaranteed constant lookup time through clever hashing and multi-entry buckets.

Table 1 explains the parameters and equations that are important in the creation of an EHT.

**Table 1:** **EHT parameters and equations**

**Symbol**	**Description**	**Effects**
*n*	number of items in table	*m*, *k*
*c*	multiplier for number of buckets	*m*, *k*
*m*=2^⌈log*cn*⌉^	number of buckets	*k*
$k = \lceil \frac {m}{n} \ln {2} \rceil $	number of hash functions/choices	num. of exp. items per bucket
*χ*	max allowed counter value	compression rate *γ*, exp. num of CAM entries
|*ω*|	on-chip mem word size [bits]	acompression rate *γ*

## Separate update and lookup engines

Previous suggestions have shown that support for updates is accompanied by enormous overhead to the tables and their summaries. The PFHT needs an additional offline BFHT to identify entries that have to be relocated. The MHT requires an occupancy bitmap and the summaries require either a deletion bitmap for lazy deletions or counting filters.

In most real-world applications, especially those that require fast lookups, updates are much rarer than lookups. By completely separating update and lookup engines, on-chip requirements can be reduced. The idea is to keep two separate summaries. One is kept online in on-chip memory and is optimized for lookups. It does not need to be exact and can be different from the update summary which is kept offline. Keeping only an approximate online summary allows individual optimization and more efficient encoding. The update engine precomputes all changes and sends modifications to the online structures. This architecture limits the applicability of the EHT to applications that are not update extensive and do not require real-time updates. That is, we buy optimized lookup performance with decreased update flexibility. That also holds for all previously mentioned summary-based hash tables as well as perfect hashing schemes. We will show that the update complexity of the EHT is comparable to that of its predecessors.

**Figure 4:**
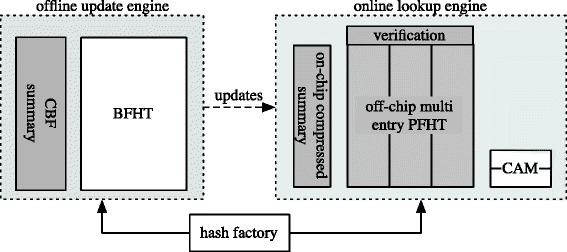
Efficient hash table design overview. The offline update engine precomputes all updates. The online lookup engine is optimized for time/space efficient lookups. The on-chip summary is not exact and compressed. The online table is pruned and provides room for multiple entries which optimized by the verification hash engine. A small extra memory is used for counter and entry overflows.

Although, some of the techniques we describe are applicable to different table and summary structures such as the FHT and MHT, we concentrate on optimizing the scheme of Song et al. 2005, which we argue has most room for improvement. Figure 4 shows a simplified overview of our design. It is relatively similar to the FHT (Song et al. 2005) with some changes in components (shaded grey in the figure). Components are an offline CBF and BFHT, an online on-chip compressed CBF summary (CCBF), the online multi-entry bucket PFHT in off-chip memory, a verifier hash engine, and a small extra memory (CAM, registers) for overflown entries (we will refer to the overflow memory as CAM in the following). Strictly, the offline CBF is not needed, the counter values could also be computed by examining the length of the linked list. However, this would lead to significant overhead when querying counters, so we keep the offline CBF for performance reasons.

### 4.1 Maximum counter value

**Figure 5:**
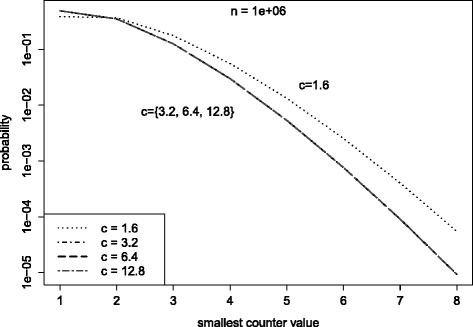
Counter value probabilities. Probability of smallest counter value in k’ counters for different c.

A lookup requires retrieving the leftmost smallest counter in the CBF summary. Successful lookup is guaranteed as long as not all counters corresponding to a key are overflown. If all the counters are overflown, it is not possible to identify the correct bucket. The goal is to identify a maximum allowed counter value *χ* where the probability that all *k*^′^<*k* chosen counters for an item equal *χ* is appropriately small. In essence, choosing an appropriate value for *χ* is a trade-off between storage saved, the number of counter overflows, and the number of expected lookup failures.

(Song et al. 2005) gives an analysis of the probability that in any *k*^′^<*k* chosen buckets the counter value has a specific height *s*. The derivation of the equation is quite complex and for simplicity left out at this point. Interested readers are referred to the actual paper. Figure 5 shows the expected smallest counter value in *k*^′^ chosen counters depending on the size *m*, or to be specific, the buckets per item constant *c*. The constant *c* is chosen to divide *m* by multiples of 2. As expected the table size has significant impact on the smallest counter value. That is, for smaller *c* the probability of choosing a higher counter is higher. When reducing *c* the maximum counter value *χ* must be higher.

To be able to retrieve all entries the event that all chosen *k*^′^<*k* counters equal *χ* must be dealt with. The easiest solution is to move entries which cannot be retrieved by calculating the counters to CAM. A small CAM must already be maintained for overflown buckets. If *χ* is chosen appropriately large the overhead is minimal.

The expected number of entries that are diverted to CAM can easily be calculated. Let *Pr*{*C*=*s*} be the probability, that of *k*^′^ chosen counters the smallest counter has value *s* and let *l* be the highest counter value to be expected in the offline summary.

(12) $$ E_{\textrm{CAM}} = \sum_{i = \chi}^{l} Pr\{ C = i\} \times n  $$

The expected number of CAM entries for *n*=10^6^,*c*={12.8,6.4,3.2,1.6,1} and *χ*={3,4,5} can be seen in Table 2. The numbers can be used as a guideline for choosing *χ*. For example, with *c*=12.8 and *χ*=3, the expected number of CAM entries is still 0. Without any additional cost, the counter-width of the summary can be reduced to 2 bits, achieving a reduction in size of 30*%*. By further providing a small CAM for few entries, *c* can be halved, leading to a summary only $\frac {1}{3}$ of the optimum in size. The trade-off improves for increasing *χ*. Consulting the numbers, each time *χ* is incremented once, *c* can be halved, at the cost of few additional CAM entries.

**Table 2:** **Expected number of CAM entries for different** ***c*** **and** ***χ*** **with** ***n*** **=10** ^**6**^ **inserted items**

	***χ***		
***c***	**5**	**4**	**3**
12.8	0	0	0
6.4	0	0	17
3.2	0	47	4183
1.6	285	5181	61110

### 4.2 Encoding

Limiting the counter range allows for better optimized encoding of the summary. We follow a simple and well known approach that is also used in (Kirsch and Mitzenmacher 2008) to pack few counters into one Byte. The difference is that we extend the scheme to an arbitrary word size to achieve higher compression rates. We argue, that SRAM, being implemented on-chip, can potentially have an arbitrary word size. Basically, the wider the memory, the more counters can be packed into one word and the more bits can be saved. In reality, one will not find memory widths >128.

Counters that are limited in range can easily be encoded in a specified number of bits. Let *ω* be a memory word, |*ω*| be its width in bits, and counters be limited to the range [0,*χ*], then the number of counters that can be packed into *ω* is defined as

(13) $$ \gamma_{p} = \left\lfloor \frac{log\ 2^{b}}{log\ |[\chi+1]| }\right\rfloor  $$

We will also refer to *γ* as the compression rate. Compression (Eq. 14) and decompression is trivial. Implemented in hardware, all counters can be unpacked in parallel.

(14) $$ \omega = \sum_{i=0}^{\gamma_{\mathrm{p}}-1} \varsigma_{i} \cdot |[\chi+1]|^{i}\  $$

We will introduce a more sophisticated Huffman compressed summary in Section 6.

### 4.3 Updates

In our design we want to completely separate updates from lookups to keep interference with the lookup process as small as possible. When performing updates, the offline table pre-computes all changes and applies them to the online CCBF, PFHT and CAM.

There are three types of entries that must be distinguished. *Offline entries* are kept in the offline BFHT. Due to overflows, each offline entry has a corresponding online entry either in the online PFHT (*table entry*) or in extra memory (*cam entry*). The update engine must be able to identify which of the *offline entries* in affected buckets are *table entries*, and which are *cam entries*. Else, it would not be possible to compute relocations without examining all possible locations in the online structure. Since we want to minimize online table access all *offline entries* are paired with a *locator*. In case the corresponding entry is a *table entry*, the *locator* is simply the index of the hash function used to store the *table entry*. If it is a *cam entry*, the locator is set to *∞*. An offline entry of item *x* thus is defined as *E*_offline_(*x*)←(*k*,*v*,*i*), where *k* denotes the key, *v* the associated value, and *i* the locator.



Algorithm 1 shows the pseudocode for insertions. First we initialize a relocation list *R*, a counter increment list *L* and an update map *M*. The list is used to collect all entries that are considered for relocation while the update map maps online buckets to their new value. The hash values for *x* are computed, counters are retrieved and the target location is identified. If all counters are equal to or exceed the maximum allowed value *χ*, the new entry must be placed into CAM and the locator is set to *∞*. Otherwise the entry’s locator is set to the index of the hash function used to store *x*. Note, that in any case we create a new offline entry with a locator set to *∞* since we cannot yet know where the item is placed. Only after relocation we can be sure, whether the item is put to the table or to CAM. We then collect all entries in affected buckets that are also either table entries or cam entries, add the new offline entry, and increment the counters. Note, that the table entries are inserted at the head of the list, while the cam entries are appended to the end. This is for balancing reasons. Online entries must be relocated prior to CAM entries since it is possible that space becomes available to hold the entries from CAM. Next the collected entries must be considered for relocation. For each collected entry we compute the hash values, the new locator and the new bucket address. We also collect all online entries for the target bucket. If the new address is different from the old address the entry *r* might be relocated. There are 3 possible events:

1. The entry is moved inside the table. *M* is updated with an empty entry at the old bucket. If the new bucket has enough space left, *M* is updated with the new bucket and *r*, else *r* must be moved to cam and *M* is updated with an *∞* bucket (indicating overflow memory) and *r*.
2. The entry is moved from cam to table. If the new bucket has enough space left, *M* is updated with {new bucket,*r*} and { *∞*, *r*}. Else *r* can’t be moved to table and *M* is not updated.
3. The entry is moved from table to cam. *M* is updated with {new bucket,0} and { *∞*, *r*}.

In any case, the locator of a relocated offline entry must be updated.

The actual update of the online structure is performed by the procedure “UpdateOnline”. The update map *M* contains bucket addresses and their associated content. The buckets in *M* are simply replaced with their new value. A special case is if bucket address is *∞*, which indicates overflow memory. In this case the overflow memory is probed for the associated entries. If the entry is present, it is removed, else it is inserted. The list *L* contains a list of counter addresses that must be incremented.

The PFHT needs to be accessed only to write changed buckets. Hence, the complexity is optimal and upper bound by the number of changed buckets. With *n* items stored in *m* buckets and $k = \frac {m}{n} \log 2$ choices, the upper bound is $O(1 + \frac {m}{n}k) = O(1 + \log 2)$. Similarly, the online CCBF needs only be accessed for counters that actually change, i.e. those that have not yet reached *χ*.

Deletions work similar to insertions with minor differences. The deleted entry *x* is removed from the offline BFHT prior to collecting entries. Then all entries in affected buckets buckets are collected and relocation computed. Afterwards, the bucket from which the item is removed is added to *M* if not already present. Then the online updates are performed. Deletions have the same complexity as insertions.

## Ignore the false positive probability

Bloom filters are usually constructed to optimize the false positive probability. In case of the MHT summaries having a negligible small false positive rate is essential to prevent type failure. In general, applications that require exact knowledge about set membership are dependent on minimizing false positives. This inevitably leads to relatively large filters.

We observe that applications using Bloom filter-based summaries as an index into another data structure, like the FHT, do not suffer from false positives, as long as a successful lookup independent of the false positive probability is guaranteed. The structure must provide a predictable worst-case lookup performance. A false positive returned by the summary leads to a table lookup that returns NULL. The worst-case performance is not affected. In conclusion, Bloom filter-based summaries can be potentially much smaller.

By reducing the address space of the summary while keeping the number of entries *n* constant, counter values and the load of buckets are expected to increase. There exists a trade-off between reducing on-chip memory requirements and the resulting counter values and bucket loads.

### 5.1 Counter values

Counter values follow a binomial distribution. With *m* possible locations and *nk* insertions (each insertion increments *k* counters) the probability *p*_*i*_ that a counter received is incremented exactly *i* times can be calculated using the following equation (Song et al. 2005).

(15) $$ p_{i} = \binom{nk}{i} \left(\frac{1}{m}\right)^{i} \left(1 - \frac{1}{m}\right)^{nk-i}  $$

**Figure 6:**
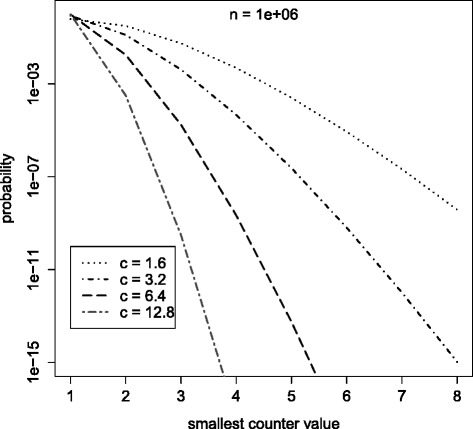
Probability of smallest counter. Counter value probabilities for different *c*. For *c*>1.6 there is no effect on the counter distribution. For *c*≤1.6 the probability for higher counters increases.

This is not entirely accurate. The probability that, due to collisions, less than *k* counters for an item can be increased, is neglected. But the estimate is close enough to allow counter value predictions. Figure 6 shows the counter distribution for different *c*. The constant *c* is chosen to divide *m* into multiples of 2. As long as *c*>1.6 the counter distribution is not affected. For *c*≤1.6 the probability for higher counters increases. This is the result of an overestimate of the number of choices *k*. Following Eq. 3, *k* depends on the number of buckets per item $\frac {m}{n}$. As $\frac {m}{n} \rightarrow 2$, *k* will lead an overestimate resulting in higher counter values. In conclusion, as long as $\frac {m}{n} > 2$ and *k* are chosen optimally, the counter values are not affected by smaller sized filters. Hence the counter width in terms of bits is unaffected.

### 5.2 Bucket load

We follow (Azar et al. 1994) to predict the expected maximum load that occurs with high probability. With *n* items, *m* buckets and *k* choices the expected maximum load is defined as

(16) $$ E_{\text{maxload}} = \frac{\ln \ln m}{\ln k}  $$

The equation holds for any *m*→*∞* with *n*=*m* and *k*≥2. In our design, however, *m*≫*n*. The result leads an overestimate of the maximum load, which in practice should be smaller. To compensate we apply the *floor* function to round to the next lower integer. A special case arises for *k*=1. This happens when $\frac {n}{m} \rightarrow 1$. Then the expected maximum load is defined as

(17) $$ E_{\textrm{maxload, k=1}} = \frac{\ln n}{\ln \ln n}  $$

Table 3 shows the expected maximum load in respect to different *c*. The results are surprisingly positive. Setting *c*=3.2 results in a summary size $\frac {1}{4}$ of the optimum proposed in (Song et al. 2005). The maximum load increases from 1 to 2 w.h.p.. In other words, allowing two entries per bucket leads to a reduction in on-chip memory size by a factor of four. The trade-off even improves for *c*=1.6. With three entries per bucket, the on-chip memory size can be reduced to $\frac {1}{8}$ of the previously suggested optimum.

**Table 3:** **Expected maximum load for different** ***c***

**c**	**k**	***E***
12.8	12	1
6.4	6	2
3.2	3	2
1.6	2	3
1	1	5

The problem arising is how to deal with more than one entry per bucket. A naive solution is to use *E* memory backs, one for each possible entry, and query them in parallel. The additional cost is acceptable compared to the saved SRAM. In Section 7 we will discuss this issue in more detail and present techniques that allow multiple entries per bucket but do not require parallel or sequential memory accesses.

## Summary compression

Section 4 introduced a simple word packing scheme for counting Bloom filters where the counters are packed in memory words. Another form of compressed counting Bloom filters has been proposed by Ficara et al. in 2008. Computing counter values in the ML-CCBF is expensive due to the fact that all preceding cells must be evaluated and the bitmaps must be accessed using perfect hash functions. These requirements render the ML-CCBF inapplicable as a summary for the EHT, since it needs to return counter multiple values on every lookup to determine the correct bucket of an item.

**Figure 7:**
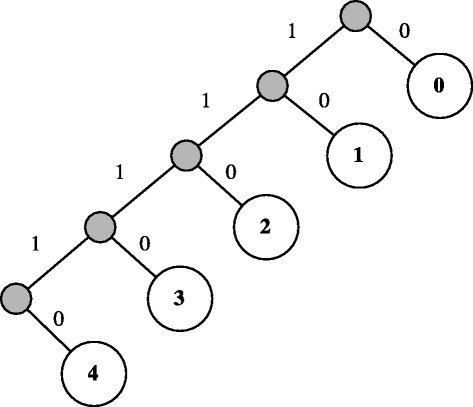
Example Huffman tree for *χ*=4.

We propose another design for compressed counting Bloom filters also based on Huffman compression, which we name *Huffman compressed counting Bloom filter (HC-CBF)*. Huffman compression is used for multiple reasons. It yields optimal and prefix free codes with the distribution of counter values. Compressed counters can be easily and individually decompressed. As mentioned in Section 4, counters are limited in range, for two reasons. First, the resulting Huffman tree is finite and very small in size. Second, the code bit-length is upper bound to the maximum allowed value *χ*+1. Figure 7 shows an example Huffman tree for *χ*=4. The tree, or codebook, can be stored in very small dedicated hardware.

To achieve real-time de-/compression the counters must be easily addressable. Storing the compressed counters consecutively is not feasible. Without the help of complex indexing structures one could not retrieve a specific value. When compressing the offline CBF we calculate the maximum number of counters *γ*_h_ that can be compressed in one memory word, such that each word encodes exactly *γ*_h_ counters. A first approach to compress the counters is shown in Algorithm 2.



The algorithm runs as long as not all counters have been processed. It iteratively tries to fit as many counters into a word *ω* as allowed by the compression rate *γ*_h_ which is initialized to *∞*. If the bit-length of *ω* would exceed the word-size, everything is reset and restarted with *γ*_h_ set to the last number of counters in *ω*. This ensures, that every word (except the last) has exactly *γ*_h_ counters encoded and allows easy indexing.

This algorithm has an obvious flaw. It depends heavily on the sequence of counters, leading to an unpredictable compression rate *γ*_h_. In addition, the compression is wasteful in storage. Since *γ*_h_ depends on the sequence of counter values, it is upper bound to the longest code sequence it can compress in one word. Assume no compression is used, then every counter will occupy three bits, which equals the length of the Huffman code for *c*=2. Thus, if during compression a long sequence of counters ≥2 is found, the compression rate *γ*_h_ will degenerate.

A better approach is to define *γ*_h_ in advance such that a desired compression rate is achieved. In general, Huffman compression only achieves improvement over word packed compression if *γ*_h_>*γ*_p_. Thus, *γ*_p_ can be used as a guideline for choosing *γ*_h_. Since we force *γ*_h_ in advance, it can lead to word overflows, if the compressed *γ*_h_ counters do not fit into a word (in the following we will refer to this scheme as *hard compression*).

Overflows can also occur during insertions. If a counter *c*<*χ*−1 is incremented and the compressed word already occupies all the available bits, then incrementing the counter will shift one bit out of the word. As a result the last counter value will not be retrievable.

There are different approaches of how to address word overflows. One is to simply ignore the affected counters and assume they have value *χ*. As long as these counters are not the smallest for any entry, the lookup process is not affected. If, however, the actual counter value is crucial to the lookup, the correct bucket of an entry can not be computed.

Alternatively, the longest code in the word could be replaced with a shorter overflow code, indicating that an overflow occurred. However, this would increase the length of nearly all counter codes and in return the probability of word overflows.

Probably the best solution is to keep a small extra memory, CAM or registers, to store the overflown bits. If counters that are completely or partially overflown must be retrieved, the remaining bits are read from the extra memory. We will show in Section 8, that depending on *γ*_h_ and *χ* the cost of additional memory is reasonably small.

With *m* counters, a compression rate of *γ* counters per word and an on-chip word-size of |*ω*| bits, the summary needs

(18) $$ \beta_{\textrm{EHT}} = \left\lceil \frac{m}{\gamma}\right\rceil \cdot |\omega |  $$

bits in total.

## Achieving deterministic lookups

A hash table bucket usually holds a single entry or a reference to a collection of entries. If more than one entry is placed in a bucket, lookup might require multiple memory reads by following pointers. This leads to more sophisticated hash table constructions that try to limit the bucket load to one with high probability.

We argue that by using intelligent hashing and wider memory a bucket can hold more than a single entry without the need of sequential or parallel memory accesses. As a preliminary, we define that a bucket will never hold reference to a collection of entries with variable size. A bucket is defined as an array of entries of fixed size, where every entry can be directly accessed.

### 7.1 Multiple entries per word

One solution is to allow more entries per memory word. Let |*ω*_D_| be the word size in bits and |*e*| be the size of an entry in bits. If |*e*|≪|*ω*_D_|, a bucket can hold up to $\left \lfloor \frac {|\omega _{\mathrm {D}}|}{|e|} \right \rfloor $ entries which can be read in one cycle. This holds for applications, like QoS/CoS classification, flow-based Server Load balancing or socket lookups, that store only small entries. But many application require larger entries (e.g. IPv6 lookup). While SRAM width is highly flexible, the word size of DRAM is usually fixed, wider memory might not be possible.

By using a hashing scheme similar to that proposed in (Bonomi et al. 2006) the size of an entry can be decreased. A class of hash functions can be used that perform transformations of the key, producing *k* digests of a fixed size, greater or equal to the size of the key. This is crucial to prevent collisions and the hash function must be collision resistant. The digest is imagined to be composed of two parts, the index to the hash table, and the verifier of the key. Let *x* be the key, *H* be the class of hash functions, [*A*] be the range of the table address space and [*V*] be the range of the remaining verifier.

(19) $$ H: U \rightarrow [A] \times [V]  $$

The verifier and the index are derived by bit-extraction. Let *h*_{0,…,*k*−1}_ be the *k* digests, then *V*(*h*_{0,…,*k*−1}_) produces the verifiers and *A*(*h*_{0,…,*k*−1}_) extracts the bucket indexes, or addresses. Instead of the key *x* only its verifier *V*(*h*_*i*_(*x*)) is stored in bucket *A*(*h*_*i*_(*x*)). To be able to identify which verifier corresponds to a given key, an identifier must be kept along the verifier, that states the hash function *i* that produced the stored verifier *V*(*h*_*i*_(*x*)). A table entry then consists of the verifier, it’s identifier (which is the index of the hash function), and the associated value *v*. Hence, *E*(*x*)←(*V*(*h*_*i*_(*x*)),*v*,*i*). The total number of bits needed is *logk*+(|*H*|−|*A*|)+|*v*| where |*y*| denotes the length of *y* in bits. Note, that the smaller |*A*| the larger |*V*|. Thus the length of the table competes with the size of the entries.

### 7.2 Multiple words per bucket

An extension to the former scheme is to allow a bucket to span multiple words. For simplicity, we assume the words are consecutive, although this is not a precondition, as long as there is a fixed offset between the words. A bucket can now be seen as a matrix of *r* entries per word and *w* words.

In addition to the address and verifier, the hash function must also lead the correct word, or row, of the bucket. Let [*W*] the range of words for each bucket.

(20) $$ H: U \rightarrow [A] \times [W] \times [V]  $$

**Figure 8:**
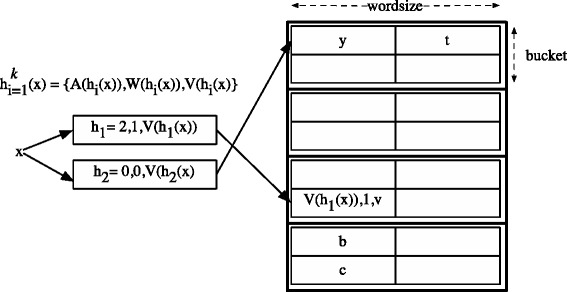
Verifier hashing and buckets with multiple entries.

Note, that in practice [*W*] will be very small, needing only 1−2 bits. Figure 8 shows the design and an example.

## Results and discussion

In this section we present and discuss results of a conceptual implementation of the EHT. The implementation is conceptual in the sense that it does *not* fully resemble the structure of the EHT but simulates it’s behavior appropriately.

Table 1 shows the parameters and equations that play a crucial role in evaluating the effects of different configurations.

For simulations we use the following values for the parameters:

$$n = \left\{10^{5}; 10^{6}\right\} ; c = \{3.2;1.6 \}; \chi = \{4;5 \}; |\omega| = \{64;128\} $$

This leads to a total of 16 different parameter configurations. The number of hash functions *k* is always chosen optimally. In the following, when referencing the parameter configurations, we will use a single hexadecimal digit *p*= [0,*F*] representing the encoding depicted in Table 4.

**Table 4:** **Parameter configurations** ***p*** **of the software simulations**

	**bit**	**3**	**2**	**1**	**0**
	**parameter**	***n***	***c***	***χ***	***|ω|***
value	0	10^5^	1.6	4	64
	1	10^6^	3.2	5	128

On each simulation we perform ten trials, that is we instantiate the EHT and fill it with *n* random keys and values. No updates are performed but the EHT is queried for all *n* and additional 2*n* random keys to verify that every key can be retrieved and to analyze the false-positive probability. As summary we use HC-CBF. The compression rate *γ*_h_ is calculated using Algorithm 2. No hard compression is used, since we want to evaluate the quality of the compression algorithm. The cost of using hard compression can be derived by examining the resulting HC-CBF and is included in the analysis.

For each try, we calculate the size of the offline CBF, the size of a CCBF and the size of the online HC-CBF. We count the frequency of all counter values in the offline summary and derive the number of overflown counters in the online summary. Every compressed word in the HC-CBF is analyzed for the number of bits that are actually used to encode counters, resulting in a histogram of code-lengths per word. In addition, the load of all online buckets is calculated and the number of CAM entries counted. Finally, we compare the on-chip requirements of the EHT with the theoretical requirements of the MHT and FHT.

### 8.1 Constant lookups

We first evaluate the performance of the EHT with respect to lookups. To achieve deterministic lookup performance, it is crucial that counter value distribution and bucket loads behave as expected. Counter distribution affects the maximum allowed counter value, which in turn affects the effectiveness of summary compression and the number of entries that have to be moved to CAM due to counter overflows.

**Figure 9:**
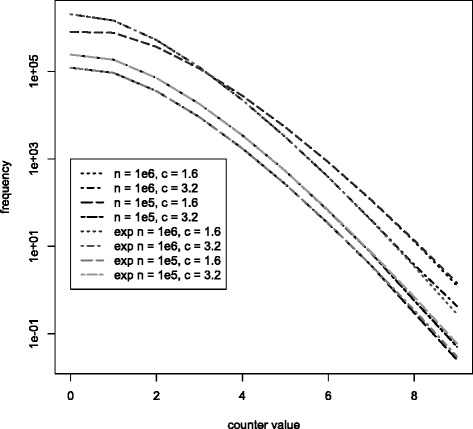
Real and expected counter frequencies.

**Counter distribution** Since the parameters *χ* and |*ω*| have no effect on the counter distribution, we count the counter frequencies for *n*={1*E*+6,1*E*+5} with sizes of *c*={1.6,3.2} and also calculate the expected frequency for each counter value. The results are shown in Figure 9. The figure shows both the expected as well as the real probabilities of counter value frequencies. The real frequencies resemble the expected frequencies almost exactly. The graphs of expected and real counter frequencies overlay up until counter value 8.

**Bucket load** The maximum load depends on the number of choices *k* and the number of items *n*. We aggregate the results of the combinations for *n* and *c* and count the number of entries in every online bucket. We then take the maximum of the frequencies to evaluate the worst-case behavior. The results are shown in Table 5.

**Table 5:** **Entry distribution and expected maximum load**

		**Load**				
***p***	***E***	**0**	**1**	**2**	**3**	**4**
0−3	3	167662	89728	5327	24	0
4−7	2	424659	99411	369	0	0
8−*B*	3	1184464	837562	80950	684	1
*C*−*F*	2	3204894	980039	10438	1	0

In the worst-case there was only a single unexpected bucket overflow, for tables with *n*=10^6^ and *c*=1.6. In all other cases no bucket overflow occurs. As long as *c*>1.6 no overflows are to be expected. Again, the experimental results resemble the theoretical assumptions.

**Overflow entries** We aggregate the results for *χ* according to *n* and *c*, calculate the average, and take the minimum/maximum values encountered. Following Eq. 12 we also calculate the expected number of CAM entries. Table 6 shows the results. On average, the number of CAM entries closely resemble the theoretical expectations. In general, only a small CAM is required. The only configurations that require a relatively large amount of CAM are the tables with *n*=10^6^ and *c*=1.6.

**Table 6:** **Real and expected number of CAM entries**

***p***	**min**	**max**	**avg**	**E**
0−1	144	209	177.95	178
2−3	2	11	6.05	6
4−5	0	1	0.15	0
6−7	0	0	0.00	0
8−9	5017	5446	5194.05	5181
*A*−*B*	236	287	258.20	265
*C*−*D*	40	61	47.00	47
*E*−*F*	0	0	0.00	0

Once again, the results closely resemble the expectations.

### 8.2 On-chip memory

We now evaluate the required on-chip memory for the EHT summary according to different parameter configurations and compare the results to related work. We consider EHT summaries with no compression (*γ*_0_), with word-packed encoding (Section 4, *γ*_p_) and with Huffman compression (Section 6, *γ*_h_).

**Compression** To analyze the achieved compression we take the minimum, maximum and average *γ*_h_ and compare that to *γ*_p_ and the number of counters if no compression is used (denoted *γ*_0_). We also include the maximum number of bits actually used to compress the counters.

The numbers in Table 7 provide a lot of useful information. With sufficiently large |*ω*| or larger *χ*, Huffman compression performs better than word packing, even without using *hard compression*. If |*ω*| is small and *χ* is also small, word packing is the better choice. In all cases, compression yields an improvement over not using compression. The counter limit *χ* only slightly influences the compression rate *γ*_h_. It’s impact on *γ*_p_ is greater by far. The reason probably is that the values for *χ* differ only by 1. It is expected that for higher differences *γ*_h_ is more affected.

**Table 7:** **Compression rate**

				***γ***_***h***_					**bits**
**n**	**c**	***χ***	***|ω|***	**min**	**max**	**avg**	***γ***_***p***_	***γ***_***0***_	**max**
10^6^	1.6	4	64	22	24	22.8	27	21.3	63.3
		5	64	21	22	21.5	24	21.3	63.3
		4	128	50	53	51.0	55	42.6	126.4
		5	128	47	51	49.5	49	42.6	125.1
	3.2	4	64	23	26	24.6	27	21.3	62.7
		5	64	24	25	24.9	24	21.3	63.2
		4	128	56	59	57.7	55	42.6	126.3
		5	128	55	58	56.9	49	42.6	126.3
10^5^	1.6	4	64	25	27	26.0	27	21.3	62.6
		5	64	24	26	25.4	24	21.3	62.5
		4	128	57	60	58.8	55	42.6	126.6
		5	128	55	60	57.8	49	42.6	125.7
	3.2	4	64	23	26	25.5	27	21.3	63.0
		5	64	23	26	24.6	24	21.3	62.1
		4	128	57	60	58.3	55	42.6	126.9
		5	128	56	59	57.0	49	42.6	125.8

**Figure 10:**
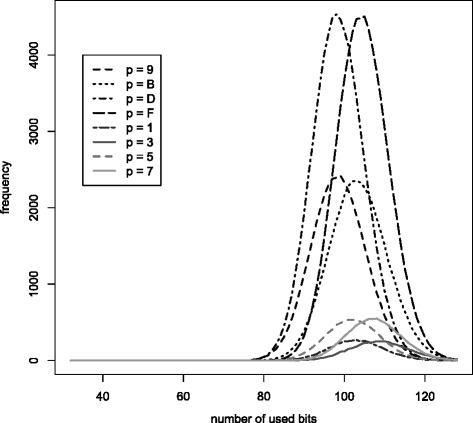
Frequencies of used bits per compressed word for |*ω*|=128.

Another interesting aspect is the frequency of used bits per word (Figure 10). The distribution follows a Poisson binomial distribution, which is to be expected. The graphs show a shift of the center depending on *χ*, which is a result of nearly equal *γ*_p_ with different code lengths. The graphs reveal potential to further reduce SRAM requirements. The compression can be improved by reducing |*ω*| while keeping the same *γ*_*h*_, thus, effectively resembling *hard compression*. For example, by reducing |*ω*| from 128 to 118 bits, 10 bits per word can be saved. Of course, this leads to a higher number of word overflows. However, making use of the frequency distribution the number of expected overflows can be kept small. By providing CAM for an additional few overflown words, some bits per on-chip memory word can be saved.

**On-chip requirement comparison** We now compare the on-chip requirements of different EHT configurations to the summaries presented in Section 2.1. None of the authors present evaluation of table sizes larger than 10^4^ entries. We are interested in much larger tables with *n*=10^6^. Thus, we calculate the expected summary sizes using the mathematical models presented in the respective original papers. Eq. 4 is used for the FHT summary. Eq. 6 provides the number of bits needed for both the MHT occupancy and deletion bitmaps. Care must be taken in choosing the parameter *c*. The original paper (Kirsch and Mitzenmacher 2005) suggests *c*=6. The later refinement (Kirsch and Mitzenmacher 2010) determines that with extensive optimization effort *c* can be smaller than two for *n*=10^4^. Unfortunately, there is no information on the lower bound for *c* with *n*=10^6^. In our calculation we therefore assume *c* to be optimal and set *c*=1. This is not a fair comparison and clearly favors the MHT but due to the lack of evidence we choose to rather be progressive. For the MHT summaries we consider both lazy deletions as well as counter-based deletions. For summaries with lazy deletions both the occupancy and deletion bitmap sizes are added to the summary size. For summaries with counters, only the size of the occupancy bitmap is added. We use Eq. 8 for SF with lazy deletions, Eq. 9 for MBF with lazy deletions, Eq. 11 for counting SF, and Eq. 10 for counting MBF. For the segmented hash (Kumar and Crowley 2005) and discriminator table (Ficara et al. 2009) we assume a fixed number of bits per item (bpi) and a linear growth. This is a realistic assumption for the Bloom filters used in segmented and peacock hash but there is no evidence that this also holds for the discriminator table. So we follow (Ficara et al. 2009) that suggests 4 bpi. For the peacock hash (Kumar et al. 2008) we assume a main table equal in size to the segmented hash above. We then calculate the size of all subtables with a scaling factor of *r*=0.1. All parameters not mentioned here are chosen optimally as suggested by the respective authors. The resulting sizes are shown in Table 8.

**Table 8:** **Comparison of on-chip requirements of different Bloom filter-based summaries for** ***n=10*** ^***6***^

**Summary**	**Size (KiB)**	**bpi**	**Eq., parameter**
FHT	6292	50.33	4, *c*=12.8, *b*=3
SF lazy	5508	44.06	6,8. *c*=1
SF counting	30023	240.18	6,11, *c*=1, *v*=2, *d*=6
MBF lazy	2742	21.93	6,9, *c*=1
MBF counting	8347	66.77	6,10, *c*=1
Segmented	2000	16	
Peacock	223	1.78	*r*=0.1
Discriminator	500	4	

Peacock hash and discriminator table clearly require the fewest bits-per-item of on-chip memory. However, Peacock hashing requires a significant amount of hashing and is not deterministic. It requires multiple sequential or parallel lookups in the worst-case, which might not be acceptable depending on the application. The discriminator table is a perfect hashing type of table that only works with static sets and is not updatable. The Segmented hash table outperforms all configurations of the MHT summaries as well as the Fast Hash Table. Of the MHT summaries, the MBF summaries are favorable over their corresponding SF summaries. The FHT resides between the SF lazy and MBF counting schemes, with lookup and update performance comparable to the MHT counting summaries. In fact, if update (especially deletion) support is important, one should rather use the FHT than the MHT.

Table 9 shows the summary sizes of all EHT configurations with *n*=10^6^ using no compression, word-based encoding, and Huffman compression. Our EHT summaries outperform all other summaries except the peacock hash and discriminator table. However, the discriminator table is not updatable and Peacock hash requires is non-deterministic. It needs multiple sequential or parallel lookups in the worst-case, which might not be acceptable depending on the application. The EHT is the only solution that guarantees a deterministic and constant lookup of *O*(1), allows near real-time updates, and still requires only a few bits per item.

**Table 9:** **On-chip requirements of EHT configurations with** ***n=10*** ^***6***^

**Configuration p**	**Uncompressed**		**Packed**		**Huffman**	
	**KiB**	**bpi**	**KiB**	**bpi**	**KiB**	**bpi**
8	787	6.29	622	4.97	737	5.89
9			611	4.88	659	5.26
A			700	5.59	781	6.24
B			685	5.48	679	5.42
C	1573	12.58	1243	9.94	1367	10.93
D			1221	9.76	1164	9.31
E			1399	11.18	1348	10.78
F			1370	10.96	1180	9.44

**Summary.** The results fully meet the expectations and backup our theoretical analysis. We have shown that our initial assumptions allow fundamental improvements over previous suggestions. Experiments have shown that the EHT performs as theoretically expected. This makes the EHT highly predictable and allows easy configuration for target applications. The effects of parameters on counter distribution, bucket load, counter and bucket overflows can easily be predicted. Evaluation shows, which hardware configurations are required for specific parameter sets. The effect of Huffman compression is much harder to predict, since all possible combinations of counter values per word would need to be predicted which is impractical. However, evaluation shows the effect of Huffman compression compared to no compression and simple word-based encoding.

In conclusion, when constructing an EHT, the following aspects must be considered.

- Reducing the length *m* is achieved by ignoring the false positive probability. As a result, bucket loads will increase which can be compensated by parallel banks, increasing the off-chip memory width or by better hashing. Analysis has shown, that the expected maximum load will not exceed 3 as long as $\frac {m}{n}>2$. Bucket overflows are extremely rare, even for a large set of items. So only a very small extra overflow memory is needed.
- By separating updates from lookups the lookup summary can be optimized for smaller size and performance. The lookup summary is not exact and limited in counter range [*χ*]. Choosing *χ* depends on the fraction $\frac {m}{n}$. Starting with *χ*=5 for $2 < \frac {m}{n} < 2.5$, *χ* can be decremented by one each time $\frac {m}{n}$ is doubled for a small overhead in terms of CAM. Performance will degrade when $\frac {m}{n} \rightarrow 2$.
- The effect of Huffman compression depends on the word-size |*ω*| and the counter limit *χ*. Word packing is favorable over Huffman compression both in complexity as well as resulting size, unless |*ω*| and *χ* are big. At the cost of few additional CAM cells, the performance of Huffman compression can be improved by reducing *ω*| while keeping *χ* constant.

Of course, the improvement in on-chip requirements is not free and is bought with additional computational complexity, wider memory and on-chip/online compression. Depending on the application and cost of hardware components, some of the suggested optimizations might not be applicable. They are, however, independent and can be easily implemented individually to optimize the total cost. A cost function can now be defined as follows. Let *α*_*S*_ be a constant cost factor of on-chip memory, *α*_*D*_ be the equivalent for off-chip memory, *w* be the width of off-chip memory in bits, *E*_*o*_ be the expected number of bucket overflows and *α*_*C*_ be the cost of CAM cells.

(21) $$ {} f_{EHT} = \alpha_{S}\, \times \,\beta_{eht} \,+\, \alpha_{D} \,\times\, (m \cdot w) \,+\, \alpha_{C} \,\times\, (E_{CAM}\,+\, E_{o}).  $$

Depending on the costs of the components the parameters for the EHT can be chosen such that the total cost is minimized.

## Conclusion

We have proven that through relaxation of requirements and exploitation of degrees of freedom on-chip memory requirements can be significantly reduced and lookup performance improved at the cost of minimal additional hardware. Based on four key ideas we have introduced new techniques to design an *Efficient Hash Table*. Our suggested improvements can be applied individually or in concert and are fully customizable to meet the requirements of the target application and hardware. The costs of each component is analyzed and evaluated and a cost function is provided that allows calculating the overall cost. The simulation results fully meet the expectations, backup our theoretical analysis and allow accurate predictions. Furthermore, we presented a thorough evaluation of the space requirements of not only multiple EHT configuration but also of its predecessors the FHT, MHT as well as segmented and peacock hash, and discriminator table in the presence of a million entries.

High amounts of on-chip memory can be traded in for comparatively small amounts of off-chip memory, additional CAM, and some computational overhead. Cleverly chosen hash functions allow the reduction of off-chip memory size. Offloading update overhead to offline structures leads to a more optimized lookup engine and allows improved encoding. We proposed two compression schemes for the summary that provide real-time performance and are easy to implement. Combined, the presented design achieves an improvement over previous solutions up to an order of magnitude, guarantees constant lookup of *O*(1), and supports near real-time updates while requiring only a few bits per item of on-chip memory.

## References

[CR1] Azar Y, Broder AZ, Karlin AR, Upfal E (1994). Balanced allocations. SIAM Journal on Computing.

[CR2] Bloom BH (1970). Space/time trade-offs in hash coding with allowable errors. Commun ACM.

[CR3] Bonomi, F, Mitzenmacher M, Panigrahy R, Singh S, Varghese G (2006) An improved construction for counting bloom filters In: Proceedings of the 14th Conference on Annual European Symposium Volume 14.. Springer. doi:10.1007/11841036_61.

[CR4] Broder AZ, Karlin AR (1990). Multilevel adaptive hashing. SODA ’90: Proceedings of the First Annual ACM-SIAM Symposium on Discrete Algorithms.

[CR5] Broder, A, Mitzenmacher M (2001) Using multiple hash functions to improve ip lookups In: INFOCOM 2001. Twentieth Annual Joint Conference of the IEEE Computer and Communications Societies. Proceedings. IEEE, 1454–14633. doi:10.1109/INFCOM.2001.916641.

[CR6] Broder, A, Mitzenmacher M (2002) Network applications of bloom filters: A survey In: Internet Mathematics, 636–646. http://www.tandfonline.com/doi/abs/10.1080/15427951.2004.10129096

[CR7] Carter LJ, Wegman MN (1977). Universal classes of hash functions. Proceedings of the Ninth Annual ACM Symposium on Theory of Computing.

[CR8] Chazelle B, Kilian J, Rubinfeld R, Tal A (2004). The bloomier filter: an efficient data structure for static support lookup tables. SODA ’04: Proceedings of the Fifteenth Annual ACM-SIAM Symposium on Discrete Algorithms.

[CR9] Cohen S, Matias Y (2003). Spectral bloom filters. SIGMOD ’03: Proceedings of the 2003 ACM SIGMOD International Conference on Management of Data.

[CR10] Dietzfelbinger M, Mehlhorn K, Rohnert H, Karlin A, Meyer auf der Heide F, Tarjan RE (1994). Dynamic perfect hashing: Upper and lower bounds. SIAM J Comput.

[CR11] Fan L, Cao P, Almeida J, Broder A (1998). Summary cache: A scalable wide-area web cache sharing protocol. Proceedings of ACM SIGCOMM.

[CR12] Ficara, D, Giordano S, Procissi G, Vitucci F (2008) Multilayer compressed counting bloom filters. INFOCOM 2008. The 27th Conference on Computer Communications. IEEE: 311–315. doi:10.1109/INFOCOM.2008.71.

[CR13] Ficara D, Giordano S, Kumar S, Lynch B (2009). Divide and discriminate: algorithm for deterministic and fast hash lookups. Proceedings of the 5th ACM/IEEE Symposium on Architectures for Networking and Communications Systems. ANCS ’09.

[CR14] Fredman ML, Komlós J, Szemerédi E (1984). Storing a sparse table with *O*(1) worst case access time. J ACM.

[CR15] Hagerup, T, Tholey T (2001) Efficient minimal perfect hashing in nearly minimal space. In: Ferreira A Reichel H (eds)STACS 2001. Lecture Notes in Computer Science, vol 2010, 317–326.. Springer. 10.1007/3-540-44693-1_28. http://dx.doi.org/10.1007/3-540-44693-1_28

[CR16] Kirsch A, Mitzenmacher M (2005). Simple summaries for hashing with multiple choices. 43rd Annual Allerton Conference on Communication, Control and Computing.

[CR17] Kirsch A, Mitzenmacher M (2008). Simple summaries for hashing with choices. IEEE/ACM Trans Netw.

[CR18] Kirsch A, Mitzenmacher M (2010). The power of one move: hashing schemes for hardware. IEEE/ACM Trans Netw.

[CR19] Kumar, S, Turner J, Crowley P (2008) Peacock hashing: Deterministic and updatable hashing for high performance networking In: INFOCOM 2008. The 27th Conference on Computer Communications, 101–105.. IEEE. doi:10.1109/INFOCOM.2008.29.

[CR20] Kumar S, Crowley P (2005). Segmented hash: an efficient hash table implementation for high performance networking subsystems. Proceedings of the 2005 ACM Symposium on Architecture for Networking and Communications Systems. ANCS ’05.

[CR21] Li, D, Chen P (2013) Summary-aided bloom filter for high-speed named data forwarding In: High Performance Switching and Routing (HPSR), 2013 IEEE 14th International Conference On, 225–226. doi:10.1109/HPSR.2013.6602321.

[CR22] Lu, Y, Prabhakar B, Bonomi F (2006) Perfect hashing for network applications In: 2006 IEEE International Symposium on Information Theory, 2774–2778.. IEEE press. http://ieeexplore.ieee.org/xpl/freeabs_all.jsp?arnumber=4036478

[CR23] Mitzenmacher MD (1996). The power of two choices in randomized load balancing. PhD thesis.

[CR24] Mitzenmacher M (2001). The power of two choices in randomized load balancing. IEEE Trans Parallel Distributed Sys.

[CR25] Mitzenmacher, M (2001b) Compressed bloom filters In: Proc. of the 20th Annual ACM Symposium on Principles of Distributed Computing. IEEE/ACM Trans. on Networking, 144–150. https://dl.acm.org/citation.cfm?id=581878

[CR26] Song H, Dharmapurikar S, Turner J, Lockwood J (2005). Fast hash table lookup using extended Bloom filter: An aid to network processing. SIGCOMM ’05.

[CR27] Vöcking B (2003). How asymmetry helps load balancing. J ACM.

